# Overexpression of CHI3L1 is associated with chemoresistance and poor outcome of epithelial ovarian carcinoma

**DOI:** 10.18632/oncotarget.5469

**Published:** 2015-10-05

**Authors:** Ying-Cheng Chiang, Han-Wei Lin, Chi-Fang Chang, Ming-Cheng Chang, Chi-Feng Fu, Tsung-Ching Chen, Shu-Feng Hsieh, Chi-An Chen, Wen-Fang Cheng

**Affiliations:** ^1^ Department of Obstetrics and Gynecology, College of Medicine, National Taiwan University, Taipei, Taiwan; ^2^ Graduate Institute of Clinical Medicine, College of Medicine, National Taiwan University, Taipei, Taiwan; ^3^ Graduate Institute of Oncology, College of Medicine, National Taiwan University, Taipei, Taiwan; ^4^ Department of Obstetrics and Gynecology, E-da Hospital, Kaohsiung, Taiwan

**Keywords:** CHI3L1, apoptosis, epithelial ovarian carcinoma, chemoresistance

## Abstract

We propose CHI3L1 as a prognostic biomarker for patients with epithelial ovarian carcinoma (EOC) and also suggest possible biological functions of CHI3L1. We measured CHI3L1 expression with quantitative real time-polymerase chain reaction (qRT-PCR) in 180 women with EOC and evaluated correlations between CHI3L1 expression, clinicopathological characteristics, and the outcomes of the patients. The expression of CHI3L1 was higher in cancerous tissues than in normal tissues. The expression of CHI3L1 was also higher in patients with a serous histological type, advanced stage, and chemoresistance. Patients with high CHI3L1 expression had a shorter progression-free survival (*p* < 0.001) and overall survival (*p* < 0.001). Patients with high CHI3L1 expression also had a high risk of recurrence (*p* < 0.001) and death (*p* < 0.001). *In vitro* studies showed that CHI3L1 up-regulated the expression of anti-apoptotic Mcl-1 protein and hampered paclitaxel-induced apoptosis of ovarian cancer cells. These results suggest that CHI3L1 shows potential as a prognostic biomarker for EOC. CHI3L1 may promote chemoresistance via inhibition of drug-induced apoptosis by up-regulating Mcl-1.

## INTRODUCTION

Epithelial ovarian carcinoma (EOC) has received increasing attention in recent years because it is associated with the highest mortality rate among gynecologic malignancies [1, 2]. The majority of patients are diagnosed at an advanced stage and have a poor prognosis [3]. Current management strategies include debulking surgery and adjuvant chemotherapy with a regimen of platinum and paclitaxel, which has a response rate of 80% for all patients and 40–60% for advanced-stage patients [4]. However, patients, especially those with an advanced stage, usually relapse after an initial response and ultimately die of recurrence [3]. Chemoresistance is an obstacle in the management of ovarian cancer. To overcome this problem several mechanisms have been proposed including suppression of apoptotic pathways, increased DNA repair and over-expression of multidrug-resistance genes [5–7]. Elucidating the mechanisms of chemoresistance may be helpful in the development of new therapeutic strategies.

Chitinase 3-like 1 (CHI3L1) is located on chromosome 1q32.1, and the product, YKL-40, a 40-kDa glycoprotein, is secreted by numerous human cells such as cartilage, synovium, endothelial cells, inflammatory cells, and cancer cells. It plays a role in cell proliferation, differentiation, apoptosis, angiogenesis, inflammation and extracellular tissue remodeling [8]. Elevated levels of serum YKL-40 are reported to be associated with several types of cancer, including ovary, breast, brain and lung cancer [9–18], and several medical and inflammatory diseases such as rheumatoid arthritis, diabetes mellitus, and coronary artery disease [19–21]. These medical, inflammatory, and malignant diseases all possibly contribute to the levels of serum YKL-40. In our study, we used real-time quantitative PCR (qRT-PCR) to measure the quantitative expression of CHI3L1 in ovarian cancer tissues without the influence of other malignancies or medical diseases.

CHI3L1 has been reported to play roles in the carcinogenesis, proliferation, invasion, and metastasis of glioma and prostate cancer [22–24]. The role of CHI3L1 in tumor angiogenesis has also been noted [25–27]. However, the role of CHI3L1 in ovarian cancer and chemoresistance is currently unknown. We initially investigated the correlation of clinical outcomes of patients and CHI3L1 expression to evaluate the feasibility of using CHI3L1 as a prognostic biomarker for patients with EOC. We used *in vitro* studies to elucidate the possible mechanism of CHI3L1 in chemoresistance. The results of this study may clarify the role of CHI3L1 in ovarian cancer and elucidate whether CHI3L1 is a potential biomarker of chemoresponse and a potential target for the treatment of patients with EOC.

## RESULTS

### The expression of CHI3L1 is higher in EOCs than in the normal ovarian tissues

**Figure 1:**
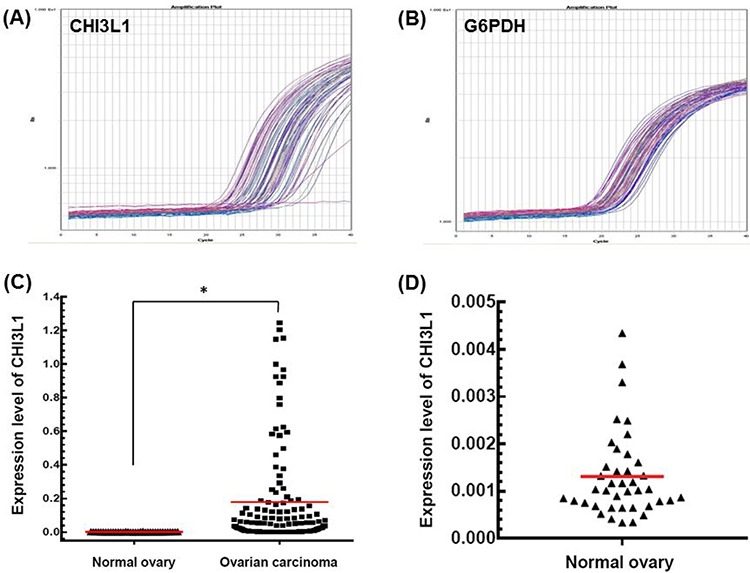
mRNA expression detected by quantitative real-time PCR **A.** Representative figure of the quantification of CHI3L1 mRNA expression in tumor tissues. **B.** Representative figure of quantification of G6PDH mRNA expression in tumor tissues. **C.** CHI3L1 mRNA expression levels between normal and cancerous ovarian tissues. Black triangles indicated the normal ovarian tissues, and black squares indicated the ovarian cancer tissues.(*p < 0.05 by the Student's *t*-test) **D.** CHI3L1 mRNA expression levels of normal ovarian tissues were shown in detail.

A total of 180 patients with EOC were enrolled and 40 normal ovarian tissues were used as a reference. The representative figures of the qRT-PCR for CHI3L1 and G6PDH are shown in Figures 1A and 1B. The mean expression of CHI3L1 in the EOCs was much higher than that in the normal ovarian tissue (0.177 vs. 0.001, *p* < 0.001, Student's *t*-test, Figure 1C and 1D).

### The expression of CHI3L1 is higher in patients with EOC that also present with a serous histologic type, advanced stage, chemoresistance, and poor outcome

The basic characteristics of the 180 patients are shown in Table 1. There were 129 (71.7%) serous carcinomas, 13 (7.2%) endometrioid carcinomas, 14 (7.8%) clear cell carcinomas, 8 (4.4%) carcinosarcomas, 3 (1.7%) transitional cell carcinomas and 13 (7.2%) carcinomas of mixed type. The expression of CHI3L1 was higher in the patients with a serous histologic type (0.174 vs. 0.029, *p* = 0.002), advanced stage (stages III/IV) (0.153 vs. 0.047, *p* = 0.046), chemoresistance (0.195 vs. 0.076, *p* = 0.004), recurrence (0.169 vs. 0.049, *p* = 0.008), and death (0.200 vs. 0.104, *p* = 0.036) than in those with a non-serous histologic type, early stage (stages I/II), chemosensitive, non recurring, and in those who were still alive (all by the Student's *t*-test) (Table 2). There was no difference in the expression of CHI3L1 between tumors with a high (III) or low (I + II) histological grade.

**Table 1:** Clinicopathological characteristics of the 180 women with EOC

	Number	Percentage (%)
Total patients	180	100
Age (years)	53.8 ± 12.2	
Histology		
Serous	129	71.7
Non-serous	51	28.3
Grade		
Low (I+II)	29	16.1
High (III)	126	70.0
Not available	25	13.9
FIGO stage		
Early (I+II)	34	18.9
Advanced (III+IV)	146	81.1
Debulking		
Optimal	96	53.3
Suboptimal	84	46.7
Platinum-based chemotherapy		
With paclitaxel	153	85.0
Without paclitaxel	27	15.0
Recurrence		
Yes	126	70.0
No	54	30.0
Death		
Yes	54	30.0
No	126	70.0
Progression-free survival (months)	10 (0–139)	
Overall survival (months)	26 (1–167)	

**Table 2:** Mean expression levels of CHI3L1 of the patients and subgroup analysis*

		Histology			Grade			FIGO stage		
	Numbers	Serous	N-serous^+^	*p* value	Low	High	*p* value	Early	Advanced	*p* value
Total patients	180	0.174	0.029	0.002	0.068	0.154	0.144	0.047	0.153	0.046
Optimal debulked patients	96	0.165	0.022	0.008	0.047	0.129	0.256	0.039	0.149	0.050
Patients with paclitaxel^#^	153	0.192	0.031	0.002	0.081	0.166	0.235	0.031	0.162	0.073

		Recurrence			Chemo-response			Outcome		
	Numbers	No	Yes	*p* value	Sensitive	Resistant	*p* value	Alive	Death	*p* value
Total patients	180	0.049	0.169	0.008	0.076	0.195	0.004	0.104	0.200	0.036
Optimally debulked patients	96	0.057	0.151	0.083	0.071	0.206	0.019	0.099	0.160	0.351
Patients with paclitaxel^#^	153	0.051	0.186	0.011	0.082	0.205	0.011	0.110	0.224	0.030

* The expression level was calculated using the 2^−ΔΔCt^ method;

+ N-serous indicated Non-serous;

# Patients with paclitaxel indicated these patients received platinum combined with paclitaxel chemotherapy; *p* value by Student's *t*-test

Of the 96 women whose residual tumor size was ≤ 1 cm after debulking surgery (Table 2), those with a serous histologic type (0.165 vs. 0.022, *p* = 0.008) or chemoresistance (0.206 vs. 0.071, *p* = 0.019) had higher levels of CHI3L1 than those with a non-serous histologic type or chemosensitivity (both by the Student's *t*-test).

Of the 153 patients who received platinum with paclitaxel chemotherapy, the levels of CHI3L1 were also higher in those with a serous histologic type (0.192 vs. 0.031, *p* = 0.002), chemoresistance (0.205 vs. 0.082, *p* = 0.011), recurrence (0.186 vs. 0.051, *p* = 0.011), and death (0.224 vs. 0.110, *p* = 0.030) than in those with a non-serous histologic type, chemosensitivity, non reoccurring, and in those who were still alive (all by the Student's *t*-test). The patients with a high grade or advanced stage did not have higher CHI3L1 levels than those with a low grade or early stage.

### CHI3L1 is a poor prognostic factor for EOC

**Figure 2:**
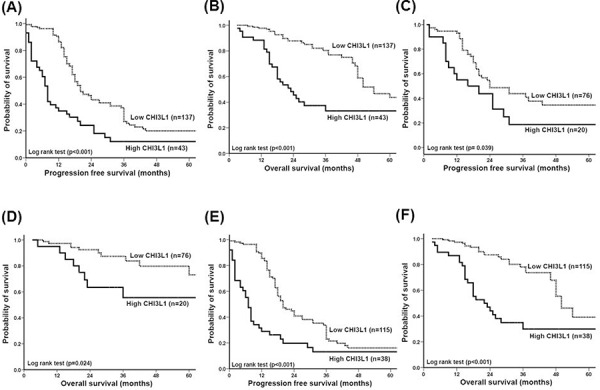
Correlation of CHI3L1 expression with progression-free survival (PFS) and overall survival (OS) of patients with ovarian cancer **A.** PFS of all 180 patients. Patients with a high CHI3L1 expression had a much shorter PFS (*p* < 0.001). **B.** OS of all 180 patients. Patients with a high CHI3L1 expression had a much, shorter OS (*p* < 0.001). **C.** PFS of the 96 patients whose residual tumor diameter was ≤ 1 cm. Patients with a high CHI3L1 expression had a shorter PFS (*p* = 0.039). **D.** OS of the 96 patients whose residual tumor diameter was ≤ 1 cm. Patients with a high CHI3L1 expression had a shorter OS (*p* = 0.024). **E.** PFS of the 153 patients who received adjuvant platinum-paclitaxel chemotherapy. Patients with a high CHI3L1 expression had a much shorter PFS (*p* < 0.001). **F.** OS of the 153 patients who received adjuvant platinum-paclitaxel chemotherapy. Patients with a high CHI3L1 expression had a much shorter OS (*p* < 0.001). All differences were calculated by the log rank test.

We further evaluated whether CHI3L1 could be a prognostic factor for the 180 patients with EOC. The patients with high CHI3L1 expression (≥ 0.1) had a shorter progression free survival (PFS) (log-rank test, *p* < 0.001, Figure 2A) and overall survival (OS) (log-rank test, *p* < 0.001, Figure 2B) than those with a low CHI3L1 expression (< 0.1) in Kaplan-Meier survival analysis.

We further analyzed the 96 patients whose tumor size was ≤ 1 cm after debulking surgery. Those with high CHI3L1 expression also had a shorter PFS (log-rank test, *p* = 0.039, Figure 2C) and OS (log-rank test, *p* = 0.024, Figure 2D) compared with those with low CHI3L1 expression. In addition, of the 153 patients who received paclitaxel-platinum chemotherapy, those with high CHI3L1 expression had a shorter PFS (log-rank test, *p* < 0.001, Figure 2E) and OS (log-rank test, *p* < 0.001, Figure 2F) compared with those with low CHI3L1 expression.

The hazard ratios (HR) of various risk factors by Cox regression analysis for the total 180 women with EOC are shown in Table 3. Using univariate analysis, high CHI3L1 expression (HR 2.32, 95% confidence interval (CI): 1.58–3.41, *p* < 0.001), advanced stage (HR: 2.46, 95% CI: 1.40 − 4.31, *p* = 0.002), high-grade (HR: 1.88, 95% CI: 1.10 − 3.20, *p* = 0.019) and residual tumor size > 1 cm after debulking surgery (HR: 1.91, 95% CI: 1.34 − 2.72, *p* < 0.001) were risk factors for recurrence. Using multivariate analysis, high CHI3L1 expression (HR: 2.91, 95% CI: 1.89 − 4.48, *p* < 0.001), advanced stage (HR: 2.47, 95% CI: 1.24 − 4.90, *p* = 0.009), high-grade (HR: 1.73, 95% CI: 1.01 − 2.97, *p* = 0.044) and residual tumor size > 1 cm after debulking surgery (HR: 1.74, 95% CI: 1.18 − 2.57, *p* = 0.005) were independent risk factors for recurrence.

**Table 3:** Cox regression model for the risk factors for recurrence and death

		Recurrence				Death			
		Univariate		Multivariate		Univariate		Multivariate	
	Numbers	HR (95% CI)	*p* value	HR (95% CI)	*p* value	HR (95% CI)	*p* value	HR (95% CI)	*p* value
**Histology**									
Non-serous	51	1.00				1.00			
Serous	129	0.68 (0.44–1.05)	0.084			1.07 (0.60–1.90)	0.800		
**FIGO stage**									
Early	34	1.00		1.00		1.00		1.00	
Advanced	146	2.46 (1.40–4.31)	0.002	2.47 (1.24–4.90)	0.009	5.20 (1.63–16.59)	0.005	4.65 (1.41–15.31)	0.011
**Grade**									
Low	29	1.00		1.00		1.00			
High	126	1.88 (1.10–3.20)	0.019	1.73 (1.01–2.97)	0.044	1.09 (0.57–2.05)	0.785		
**Debulking surgery**									
Optimal	96	1.00		1.00		1.00		1.00	
Suboptimal	84	1.91 (1.34–2.72)	< 0.001	1.74 (1.18–2.57)	0.005	3.09 (1.81–5.26)	< 0.001	2.95 (1.70–5.10)	< 0.001
**Platinum-based chemotherapy**									
Without paclitaxel	27	1.00				1.00			
With paclitaxel	153	1.30 (0.79–2.15)	0.296			1.71 (0.81–3.61)	0.156		
**CHI3L1 expression**									
Low	137	1.00		1.00		1.00		1.00	
High	43	2.32 (1.58–3.41)	< 0.001	2.91 (1.89–4.48)	< 0.001	3.03 (1.84–5.00)	< 0.001	4.03 (2.37–6.87)	< 0.001

Using univariate analysis, high CHI3L1 expression (HR 3.03, 95% CI: 1.84 − 5.00, *p* < 0.001), advanced stage (HR: 5.20, 95% CI: 1.63 − 16.59, *p* = 0.005) and residual tumor size > 1 cm after debulking surgery (HR: 3.09, 95% CI: 1.81 − 5.26, *p* < 0.001) were risk factors for death. However, multivariate analysis showed that high CHI3L1 expression (HR: 4.03, 95% CI: 2.37 − 6.87, *p* < 0.001), advanced stage (HR: 4.65, 95% CI: 1.41 − 15.31, *p* = 0.011) and residual tumor size > 1 cm after debulking surgery (HR: 2.95, 95% CI: 1.70 − 5.10, *p* < 0.001) were independent risk factors for death.

These results suggest that CHI3L1 expression is a poor prognostic factor for patients with EOC.

### CHI3L1 inhibits apoptosis of human ovarian and endometrial cancer cells treated with cytotoxic drugs

**Figure 3:**
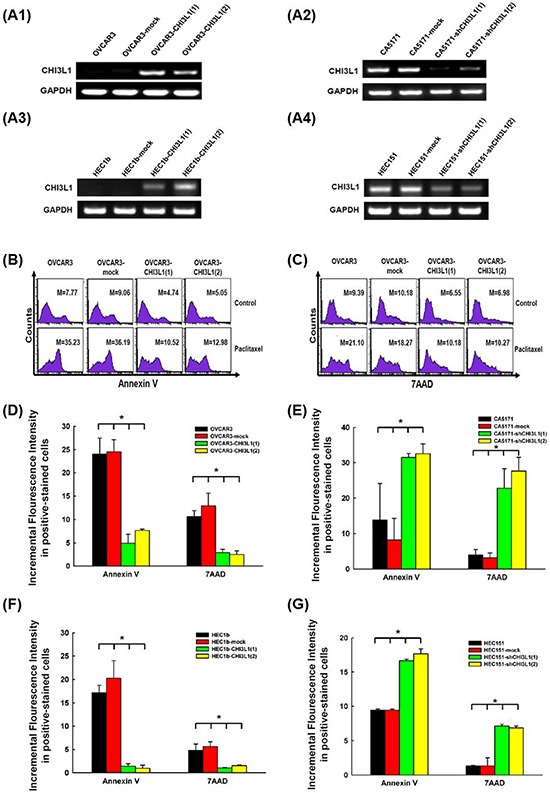
*In vitro* apoptotic assays of ovarian cancer (OVCAR3, CA5171) and endometrial cancer (HEC1b, HEC151) original cells, and their transfectants treated with respective cytotoxic drug **A.** RT-PCR of CHI3L1 expression in the various cell lines. A1: The expression of CHI3L1 was higher in the OVCAR3-CHI3L1 transfectants than those in the original OVCAR3 and mock-transfectants. A2: The expression of CHI3L1 was lower in the CA5171-shCHI3L1 transfectants than those in the original CA5171 and mock-transfectants. A3: The expression of CHI3L1 was higher in the HEC1b-CHI3L1 transfectants than those in the original HEC1b and mock-transfectants. A4: The expression of CHI3L1 was lower in the HEC151-shCHI3L1 transfectants than those in the original HEC151 and mock-transfectants. **B.** Representative figures of flow cytometric analysis for annexin V-staining in original and various OVCAR3 transfectants treated with paclitaxel. **C.** Representative figures of flow cytometric analysis for 7AAD-stained in original and various OVCAR3 transfectants treated with paclitaxel. (The M values in (B) and (C) indicated the median value of fluorescence intensity of all cells stained with annexin V or 7AAD in original and various OVCAR3 transfectants). **D.** Bar figures of the incremental fluorescence intensity of Annexin V and 7AAD -positive cells in original and various OVCAR3 transfectants treated with paclitaxel for 48 hours (*p < 0.05 by ANOVA). The incremental fluorescence intensities of annexin V and 7AAD in the various OVCAR3 CHI3L1 transfectants were significantly lower than those in the original OVCAR3 and mock-transfected OVCAR3 cells. **E.** Bar figures of the incremental fluorescence intensity of Annexin V and 7AAD -positive cells in original and various CA5171 transfectants treated with paclitaxel for 48 hours (*p < 0.05 by ANOVA). The incremental fluorescence intensities of annexin V and 7AAD in the various CA5171 shCHI3L1 transfectants were higher than those in the original CA5171 and mock-transfected CA5171 cells. **F.** Bar figures of the incremental fluorescence intensity of Annexin V and 7AAD -positive cells in original and various HEC1b transfectants treated with paclitaxel for 48 hours (*p < 0.05 by ANOVA). The incremental fluorescence intensities of annexin V and 7AAD in the various HEC1b CHI3L1 transfectants were lower than those in the original HEC1b and mock-transfected HEC1b cells. **G.** Bar figures of the incremental fluorescence intensity of Annexin V and 7AAD -positive cells in original and various HEC151 transfectants treated with paclitaxel for 48 hours (*p < 0.05 by ANOVA). The incremental fluorescence intensities of annexin V and 7AAD in the various HEC151 shCHI3L1 transfectants were higher than those in the original HEC151 and mock-transfected HEC151 cells.

To investigate the possible biological effects of CHI3L1 on ovarian cancer cells, CHI3L1-transfected OVCAR3 cells were generated for *in vitro* apoptosis-related assays. The RNA transcription levels of CHI3L1 in various OVCAR3 CHI3L1 transfectants were higher than those in the mock-transfected and original OVCAR3 cells (Figure 3A1). The representative figures of flow cytometric analysis for the detection of annexin V-positive and 7AAD-positive cells in various OVCAR3 transfectants treated with paclitaxel are shown in Figures 3B and 3C. The incremental fluorescence intensity of annexin V in the various OVCAR3 CHI3L1 transfectants was much lower than those in the original OVCAR3 and mock-transfected OVCAR3 cells (original OVCAR3: 24.01 ± 3.45, OVCAR3-mock: 24.53 ± 2.60, OVCAR3-CHI3L1(1):4.91±1.96, OVCAR3-CHI3L1(2): 7.64 ± 0.29, ANOVA test, *p* = 0.001, Figure 3D), when treated with paclitaxel for 48 hours. The incremental fluorescence intensity of 7AAD in the OVCAR3 CHI3L1 transfectants was also much lower than those in the original OVCAR3 and mock-transfected OVCAR3 cells (original OVCAR3: 10.63 ± 1.27, OVCAR3-mock: 12.91 ± 2.71, OVCAR3-CHI3L1(1): 2.91 ± 0.72, OVCAR3-CHI3L1(2): 2.49 ± 0.79, ANOVA test, *p* = 0.003, Figure 3D), when treated with paclitaxel for 48 hours. However, there were no significant differences in the incremental fluorescence intensity of annexin V (original OVCAR3: 6.66 ± 0.26, OVCAR3-mock: 6.90 ± 0.10, OVCAR3-CHI3L1(1): 9.24 ± 1.41, OVCAR3-CHI3L1(2): 9.97 ± 0.90, ANOVA test, *p* = 0.11) or 7AAD (original OVCAR3: 0.62 ± 0.35, OVCAR3-mock: 0.95 ± 0.06, OVCAR3-CHI3L1(1): 1.25 ± 0.11, OVCAR3-CHI3L1(2): 0.94 ± 0.01, ANOVA test, *p* = 0.27) in the cell lines treated with cisplatin.

The CHI3L1-knockdown CA5171 cells were generated for *in vitro* apoptosis-related assays. The RNA transcription levels of CHI3L1 in various CA5171 shCHI3L1 transfectants were lower than those in the mock-transfected and original CA5171 cells (Figure 3A2). The incremental fluorescence intensity of annexin V in the various CA5171 shCHI3L1 transfectants was much higher than those in the original CA5171 and mock-transfected CA5171 cells (original CA5171: 13.85 ± 10.26, CA5171-mock: 8.24 ± 6.06, CA5171-shCHI3L1(1): 31.54 ± 1.14, CA5171-shCHI3L1(2): 32.62 ± 2.75, ANOVA test, *p* = 0.006, Figure 3E), when treated with paclitaxel for 48 hours. The incremental fluorescence intensity of 7AAD in the CA5171 shCHI3L1 transfectants was also higher than those in the original CA5171 and mock-transfected CA5171 cells (original CA5171: 3.96 ± 1.57, CA5171-mock: 3.16 ± 1.39, CA5171-shCHI3L1(1): 22.77 ± 5.54, CA5171-shCHI3L1(2): 27.65 ± 3.92, ANOVA test, *p* = 0.025, Figure 3E), when treated with paclitaxel for 48 hours. However, there were no significant differences in the incremental fluorescence intensity of annexin V (original CA5171: 1.40 ± 0.49, CA5171-mock: 0.95 ± 0.97, CA5171-shCHI3L1(1): 2.03 ± 0.59, CA5171-shCHI3L1(2): 3.66 ± 1.66, ANOVA test, *p*=0.34) or 7AAD (original CA5171: 1.97 ± 1.08, CA5171-mock: 2.06 ± 1.17, CA5171-shCHI3L1(1): 1.52 ± 0.46, CA5171-shCHI3L1(2): 3.44 ± 1.28, ANOVA test, *p* = 0.62) in the cell lines treated with cisplatin. These results suggest that the CHI3L1 can inhibit the paclitaxel-induced apoptosis of human ovarian cancer cells.

To confirm if these biological effects of CHI3L1 can be also identified on endometrial cancer cells, CHI3L1-transfected HEC1b cells were generated for *in vitro* apoptosis-related assays. The RNA transcription levels of CHI3L1 in various HEC1b CHI3L1 transfectants were higher than those in the mock-transfected and original HEC1b cells (Figure 3A3). The incremental fluorescence intensity of annexin V in the various HEC1b CHI3L1 transfectants was much lower than those in the original HEC1b and mock-transfected HEC1b cells (original HEC1b: 17.17 ± 0.91, HEC1b-mock: 20.26 ± 2.17, HEC1b-CHI3L1(1): 1.37 ± 0.30, HEC1b-CHI3L1(2): 0.94 ± 0.38, ANOVA test, *p* < 0.001, Figure 3F), when treated with paclitaxel for 48 hours. The incremental fluorescence intensity of 7AAD in the HEC1b CHI3L1 transfectants was also lower than those in the original HEC1b and mock-transfected HEC1b cells (original HEC1b: 4.83 ± 1.34, HEC1b-mock: 5.64 ± 0.98, HEC1b-CHI3L1(1): 1.02 ± 0.04, HEC1b-CHI3L1(2): 1.51 ± 0.12, ANOVA test, *p* = 0.009, Figure 3F), when treated with paclitaxel for 48 hours. However, there were no significant differences in the incremental fluorescence intensity of annexin V (original HEC1b: 4.77 ± 3.51, HEC1b-mock: 4.56 ± 3.52, HEC1b-CHI3L1(1): 2.59 ± 1.06, HEC1b-CHI3L1(2): 3.42 ± 0.77, ANOVA test, *p* = 0.93) or 7AAD (original HEC1b: 1.08 ± 2.44, HEC1b-mock: 2.02 ± 1.91, HEC1b-CHI3L1(1): 2.04 ± 1.44, HEC1b-CHI3L1(2): 1.88 ± 1.20, ANOVA test, *p* = 0.24) in the cell lines treated with cisplatin.

The CHI3L1-knockdown HEC151 cells were generated for *in vitro* apoptosis-related assays. The RNA transcription levels of CHI3L1 in various HEC151 shCHI3L1 transfectants were lower than those in the mock-transfected and original HEC151 cells (Figure 3A4). The incremental fluorescence intensity of annexin V in the various HEC151 shCHI3L1 transfectants was much higher than those in the original HEC151 and mock-transfected HEC151 cells (original HEC151: 9.44 ± 0.16, HEC151-mock: 9.42 ± 0.17, HEC151-shCHI3L1(1): 16.68 ± 0.20, HEC151-shCHI3L1(2): 17.68 ± 0.72, ANOVA test, *p* < 0.001, Figure 3G), when treated with paclitaxel for 48 hours. The incremental fluorescence intensity of 7AAD in the HEC151 shCHI3L1 transfectants was also higher than those in the original HEC151 and mock-transfected HEC151 cells (original HEC151: 1.34 ± 0.07, HEC151-mock: 1.34 ± 1.18, HEC151-shCHI3L1(1): 7.14 ± 0.25, HEC151-shCHI3L1(2): 6.87 ± 0.27, ANOVA test, *p* = 0.004, Figure 3G), when treated with paclitaxel for 48 hours. However, there were no significant differences in the incremental fluorescence intensity of annexin V (original HEC151: 1.14 ± 0.10, HEC151-mock: 1.21 ± 0.19, HEC151-shCHI3L1(1): 0.72 ± 0.21, HEC151-shCHI3L1(2): 0.43 ± 0.25, ANOVA test, *p*=0.13) or 7AAD (original HEC151: 4.94 ± 2.72, HEC151-mock: 4.01 ± 1.77, HEC151-shCHI3L1(1): 4.46 ± 1.46, HEC151-shCHI3L1(2): 5.59 ± 0.68, ANOVA test, *p* = 0.93) in the cell lines treated with cisplatin. These results suggest that the CHI3L1 can inhibit the paclitaxel-induced apoptosis of human endometrial cancer cells.

### CHI3L1 inhibits apoptosis of human ovarian and endometrial cancer cells by up-regulating anti-apoptotic Mcl-1

**Figure 4:**
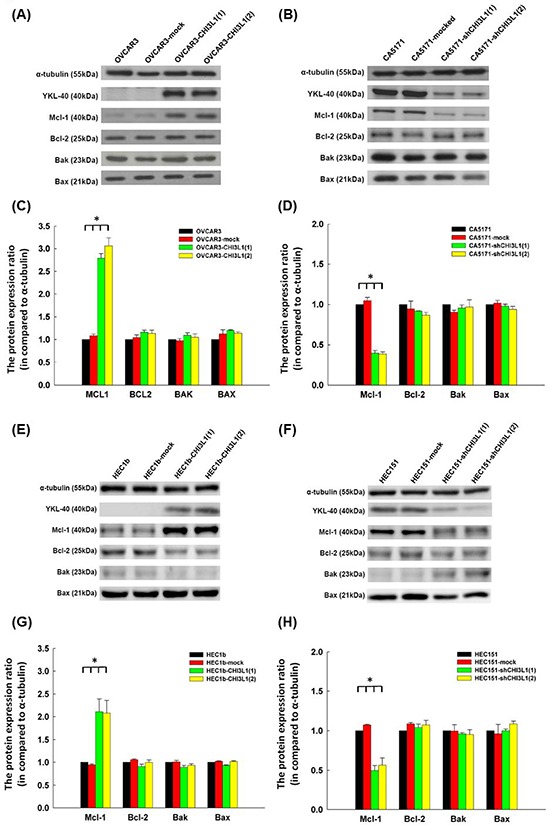
Western blot analysis of ovarian cancer (OVCAR3, CA5171) and endometrial cancer (HEC1b, HEC151) original cells and their transfectants **A.** Representative figures of Western blots of apoptosis-related molecules in OVACR3 original cells and their transfectants. **B.** Representative figures of Western blots of apoptosis-related molecules in CA5171 original cells and their transfectants. **C.** Bar figures of protein expressions of various molecules in OVACR3 original cells and their transfectants. The expressions of Mcl-1 increased significantly in the CHI3L1-transfected OVCAR3 cells (*p < 0.05 by ANOVA). **D.** Bar figures of protein expressions of various molecules in CA5171 original cells and their transfectants. The expressions of Mcl-1 decreased significantly in the shCHI3L1-transfected CA5171 cells (*p < 0.05 by ANOVA). **E.** Representative figures of Western blots of apoptosis-related molecules in HEC1b original cells and their transfectants. **F.** Representative figures of Western blots of apoptosis-related molecules in HEC151 original cells and their transfectants. **G.** Bar figures of protein expressions of various molecules in HEC1b original cells and their transfectants. The expressions of Mcl-1 increased significantly in the CHI3L1-transfected HEC1b cells (*p < 0.05 by ANOVA). **H.** Bar figures of protein expressions of various molecules in HEC151 original cells and their transfectants. The expressions of Mcl-1 decreased significantly in the shCHI3L1-transfected HEC151 cells (*p < 0.05 by ANOVA).

We evaluated whether CHI3L1 could regulate apoptosis-related molecules. The representative figures of the expressions of various apoptosis-related molecules, including Mcl-1, Bcl-2, Bak and Bax, were detected by immunoblotting in OVCAR3 and CA5171 original cells (Figures 4A and 4B). The Mcl-1 levels in the OVCAR3 CHI3L1 transfectants were much higher than in the original and mock-transfected OVCAR3 cells (original OVCAR3: 1.0, OVCAR3-mock: 1.09 ± 0.03, OVCAR3-CHI3L1(1): 2.79 ± 0.18, OVCAR3-CHI3L1(2): 3.06 ± 0.16, ANOVA test, *p* < 0.001, Figure 4C). There were no differences in protein levels of the other apoptosis-related molecules.

However, Mcl-1 levels in the CA5171 shCHI3L1 transfectants were much lower than in the original and mock-transfected CA5171 cells (original CA5171: 1.0, CA5171-mock: 1.05 ± 0.04, CA5171-shCHI3L1(1): 0.40 ± 0.03, CA5171-shCHI3L1(2): 0.39 ± 0.03, ANOVA test, *p* < 0.001, Figure 4D). There were no differences in protein levels of the other apoptosis-related molecules. These results indicate that CHI3L1 may inhibit apoptosis by enhancing the expression of Mcl-1 in ovarian cancer cells.

The representative figures of the expressions of various apoptosis-related molecules, including Mcl-1, Bcl-2, Bak and Bax, detected by immunoblotting in HEC1b and HEC151 original cells and their transfectants are shown in Figures 4E and 4F. The Mcl-1 levels in the HEC1b CHI3L1 transfectants were higher than in the original HEC1b and mock-transfected HEC1b cells (original HEC1b: 1.0, HEC1b-mock: 0.94 ± 0.02, HEC1b-CHI3L1(1): 2.11 ± 0.28, HEC1b-CHI3L1(2): 2.08 ± 0.28, ANOVA test, *p* = 0.004, Figure 4G). There were no differences in protein levels of the other apoptosis-related molecules.

However, the Mcl-1 levels in the HEC151 shCHI3L1 transfectants were much lower than in the original HEC151 and mock-transfected HEC151 cells (original HEC151: 1.0, HEC151-mock: 1.07 ± 0.01, HEC151-shCHI3L1(1): 0.49 ± 0.06, HEC151-shCHI3L1(2): 0.56 ± 0.09, ANOVA test, *p* < 0.001, Figure 4H). There were no differences in the protein levels of the other apoptosis-related molecules. These results indicate that CHI3L1 may inhibit apoptosis by enhancing the expression of Mcl-1 in endometrial cancer cells.

## DISCUSSION

Several studies have been published on the ability of serum or plasma YKL-40 to detect and assess therapeutic responses or as a prognostic predictor of ovarian cancer [9–15], although other studies have not supported this role [28, 29]. Elevated levels of serum YKL-40 have been reported in several other types of cancer, including breast [16], gastrointestinal tract [30], prostate [31], brain [17], and lung [18] cancer. In addition, several medical and inflammatory diseases have been associated with elevated serum levels of YKL-40, including polycystic ovarian syndrome [32], rheumatoid arthritis [19], diabetes mellitus [20], stroke [33], coronary artery disease [21] and asthma [34]. Only a few studies have investigated YKL-40 expression in ovarian cancer tissues using immunohistochemistry, and the results have not been promising [14, 35, 36]. Therefore, we used qRT-PCR in this study to quantitatively measure CHI3L1 expression in ovarian cancer tissues without the influence of other malignancies or medical diseases.

In this study, we found that CHI3L1 expression in cancerous tissues is a prognostic biomarker for patients with EOC. CHI3L1 expression was higher in patients with a serous histological type, advanced stage, chemoresistance and poor prognosis. Previous studies of ovarian cancer tissue also showed that YKL-40 was related to histology, stage and shorter survival by immunohistochemistry [14, 35, 36]. A high CHI3L1 expression was associated with recurrence and death in our study. The correlation of poor outcome and high CHI3L1 expression was also observed in patients whose residual tumor size was ≤ 1 cm after debulking surgery or chemotherapy with paclitaxel. In multivariate Cox regression analysis, we found that high CHI3L1 expression, advanced stage, and residual tumor size > 1 cm after debulking surgery were independent risk factors for recurrence and death. Therefore, we further investigated whether CHI3L1 plays a role in the chemoresistance of ovarian carcinoma.

Studies have found possible biological functions and mechanisms for CHI3L1 in cancer cells [22, 37]. For example, CHI3L1 regulates the proliferation of glioma cells through MAPK and AKT pathways [37], and it also plays roles in tumorigenesis and local invasion through matrix metalloproteinase-2 [22]. Nuclear factor I-X3 and STAT3 has also been identified in controlling the migration and invasion of glioma cells via the regulation of CHI3L1 [24]. CHI3L1 has also been found to promote tumor angiogenesis by inducing the coordination of membrane-bound receptor syndecan-1 and integrin alpha(v)beta(3), and by activating MAPK kinase1/2 in endothelial cells [25, 26]. However, the biological function of CHI3L1 in ovarian cancer had not been explored.

We found that CHI3L1 interfered with the paclitaxel-induced apoptosis of human ovarian cancer cells by up-regulating the anti-apoptotic Mcl-1 molecule. The cell toxicity of paclitaxel acts through the stabilization of microtubules, cell-cycle arrest in the G2/M-phase and the activation of apoptotic pathways [38]. Several mechanisms of drug resistance have been proposed, including suppression of apoptotic pathways [5], increased DNA repair [6] and over-expression of multidrug-resistance genes [7]. Our results indicate that CHI3L1 could up-regulate the expression of Mcl-1 in favor of anti-apoptotic pathways to hamper the apoptosis induced by chemotherapeutic drugs in ovarian cancer cells. However, we did not find an anti-apoptotic effect of CHI3L1 in the ovarian cancer cells treated with cisplatin. OVCAR3 and CA5171 cells are resistant to cisplatin [39, 40], and this may have a determinative effect on evaluating the role of CHI3L1 in cisplatin-induced apoptosis. We also confirmed these findings in endometrial cancer cell models. Further studies are needed to uncover the complex mechanism of cisplatin- or paclitaxel-induced apoptosis involving CHI3L1 and apoptosis-regulatory molecules.

The primary treatments for epithelial ovarian carcinoma include cytoreduction surgery and adjuvant chemotherapy, however survival is affected by disease recurrence and chemoresistance [41]. The goal of primary cytoreduction surgery is to remove as much tumor mass as possible to prevent residual disease, however it has been reported that maximal cytoreduction has only been achieved in 20–90% of patients, depending on the experience of the surgeon [42]. Chemoresistance is another problem in the management of ovarian cancer, for which several novel agents are now under investigation [3, 41, 43]. Although recurrent disease is generally incurable, management with surgery, combination of salvage chemotherapy, and targeted therapy might improve progression-free survival [41]. Advances in the molecular biology of ovarian cancer can promote the development of targeted therapy to provide personalized medicine. Our study indicated that a high CHI3L1 expression was an independent prognostic factor for EOC, even in the patients whose residual tumor size < 1cm after debulking surgery or who receiving paclitaxel-platinum chemotherapy. Our *in vitro* experiments also show that CHI3L1 can promote ovarian cancer cell resistance to paclitaxel by up-regulating Mcl-1. These findings may be incorporated into trials of new agents to treat EOC. Based on the CHI3L1 expression in ovarian cancer tissue, appropriate candidates can be selected to receive combinations of paclitaxel-platinum chemotherapy and other novel anti-apoptotic agents, especially Mcl-1. A combination of anti- CHI3L1 neutralizing antibody and irradiation can synergistically inhibit tumor vascularization and progression in xenografted brain tumor models [44, 45]. Also, resveratrol was found to repress the expression of CHI3L1 in glioma cells in *in vitro* experiments [46]. Currently, no cancer therapeutic agent related to CHI3L1 or YKL 40 has been reported. Our current study may provide preliminary findings for developing specific therapies [47]. Further clinical trials are needed to prove the utility of CHI3L1 in managing patients with ovarian cancer.

## MATERIALS AND METHODS

### Patients and specimens

The study protocol was approved by the Institutional Review Board of the hospital. Patients with EOC who received staging or debulking surgery and adjuvant platinum-based chemotherapy were enrolled, and informed consent was obtained before collecting samples. Cancerous tissue specimens were collected during surgery, immediately frozen in liquid nitrogen, and stored at −70°C until the experiments. Normal ovarian tissues were collected from women with benign gynecologic lesions who underwent bilateral salpingo-oophorectomy and were also immediately frozen in liquid nitrogen and stored at −70°C until the experiments. The maximal residual tumor size after surgery was recorded and was divided as two groups- ≤ 1 cm and > 1 cm. Histological grading was based on the International Union against Cancer criteria, and staging was based on the criteria of the International Federation of Gynecology and Obstetrics [48]. The patients received regular follow-up every 3 months after the primary treatment. Abnormal results of imaging studies (including computerized tomography or magnetic resonance imaging), elevated CA125 (more than 2 times the upper normal limit) of two consecutive tests in 2-week intervals, or biopsy-proven disease were defined as recurrence. Patients with disease progression or recurrence 6 months or less after completing adjuvant chemotherapy were defined as being chemoresistant, while those without recurrence or recurrence more than 6 months after completing adjuvant chemotherapy were defined as chemosensitive. Progression-free survival was calculated as the period from the date of completing chemotherapy to the date of confirmed recurrence, disease progression, or last follow-up. Overall survival was calculated as the period from surgery to the date of death associated with disease or the date of last follow-up. Pre-existing clinical data including age, menopausal status, clinical stage, treatment history, surgical findings during debulking, recurrence status, and survival were collected from medical records stored in a centralized database.

### RNA extraction from cancer tissues

Total RNA of the tissue specimens was isolated with Trizol reagent (Invitrogen Corporation, Carlsbad, CA), according to the manufacturer's instructions. The samples were subsequently passed over a Qiagen RNeasy column (Qiagen, Valencia, CA) for removal of small fragments that affect RT reaction and hybridization quality. After RNA recovery, cDNA was synthesized by a chimeric oligonucleotide with an oligo-dT and a T7 RNA polymerase promoter at a concentration of 100 pmol/μL.

### Quantitative real-time polymerase chain reaction (QRT-PCR)

First-strand cDNA was synthesized using a RevertAid first strand cDNA synthesis kit (Thermo Fisher Scientific, Waltham, MA). QRT-PCR was performed using a LightCycler Real-Time detection system (Roche Diagnostics, Mannheim, Germany). The relative abundance of mRNA level was calculated by using the comparative method with G6PDH as the internal control. Detection of CHI3L1 was performed using the primer Hs00609691_m1 (TaqMan® Assays, Life Technologies Corporation) with 50 cycles of 10 seconds at 95°C, 10 seconds at 60°C, and 10 seconds at 72°C. Detection of G6PDH was performed using the LightCycler h-G6PDH housekeeping gene set (Roche Applied Science, Indianapolis, IN) with 50 cycles of 10 seconds at 95°C, 15 seconds at 55°C, and 15 seconds at 72°C.

The comparative 2^−ΔΔCt^ method was used to calculate the expression of the target gene as described previously [49]. Generation of quantitative data was based on the number of cycles needed for amplification-generated fluorescence to reach a specific threshold of detection (the Ct value). For the relative quantification of gene expression on the basis of adding fixed amounts of RNA-starting material to the reactions, the Ct values obtained for each real-time PCR were first transformed using the term E^−Ct^, where E is reaction efficiency, divided by the corresponding value obtained for the same gene in the reference sample (normal ovarian tissues). The following equation was used to calculate the expression level of CHI3L1 in each sample: Relative expression level of CHI3L1 = 2^−ΔΔCt^, ΔCt = Ct _target (CHI3L1)_ − Ct _housekeeping (G6PDH),_ ΔΔCt = ΔCt _sample (ovarian cancer tissue)_ −ΔCt _calibrator (normal ovarian tissue)_.

### Reverse-transcription polymerase chain reaction (RT-PCR)

RNA was first reverse-transcribed to cDNA using a Moloney murine leukemia virus reverse transcriptase kit (Invitrogen Life Technologies, San Diego, CA). To generate CHI3L1, sense primer TGTGAAGGCGTCTCAAACAG and anti-sense primer AATTCGGCCTTCATTTCCTT were used for 30 cycles. Glyceraldehyde-3-phosphate dehydrogenase (GAPDH) was used as the housekeeping gene to compare with CHI3L1. To generate GAPDH, sense primer ACCCAGAAGACTGTGGATGG and antisense promer TGCTGTA GCCAAATTCGTTG were used for 30 cycles. The PCR products were then analyzed in 1% agarose gel with EtBr staining in TBE solution.

### Transfection of CHI3L1 in OVCAR3 cells

The OVCAR3 cell line was obtained from the American Type Culture Collection (Manassas, VA). The transcription status of CHI3L1 in OVCAR3 cells was checked by RT-PCR as described above. The plasmid PLKO_AS3w.puro was purchased from the National RNAi Core Facility (Academia Sinica, Taiwan). To generate PLKO_AS3w CHI3L1.puro, CHI3L1 was first amplified by PCR using OVCAR3 cell cDNA as the template with sense primer (NheI) Hu-CHI3L1_S_NheI GGCTAGCACCATGGGTGTGAAGGCGTCTCAAAC and anti-sense primer (PstI) Hu-CHI3L1_AS_PstI TAACTGCAGCTCAGCCTGGGACTCAGCA. The amplified product was then cloned into the NheI/PstI sites of PLKO_AS3w.puro. The transfection was followed the protocol on the website http://rnai.genmed.sinica.edu.tw/webContent/web/protocols. OVCAR3 cells transfected with PLKO_AS3w.puro (mock) were used as the control. The CHI3L1 expression was assayed by RT-PCR and Western blot. The stable clones of various OVCAR3 transfectants were used in the following experiments.

### Knock-down of CHI3L1 in CA5171 cells

To eliminate endogenous CHI3L1 expression in CA5171 cells [40], an *in-vitro* knock-down experiment by small hairpin RNA (shRNA) targeting to CHI3L1 were performed. shRNA for CHI3L1 was purchased from National RNAi Core Facility. CHI3L1 shRNA constructed lentivirus were collected from 293T cells transfected with shRNA vector, Δ8.91and pMD.G. All plasmids were re-suspended in Opti-MEM® medium and transfected by using Lipofectamine2000 reagent according to the manufacturer's instructions. To generate CHI3L1-knockdown CA5171 cells (CA5171-shCHI3L1), CA5171 cell were seeded in 6-cm dishes and infected with CHI3L1 shRNA lentivirus containing 10 μg/ml polybrene for 48 hours. For selecting of stable clones, puromycin were added to the culture medium for further 48-hour incubation. The CHI3L1 expression was assayed by RT-PCR and Western blot. The stable clones of various CA5171 transfectants were used in the following experiments.

### Transfection of CHI3L1 in HEC1b cells

The HEC1b cell line was obtained from the American Type Culture Collection (Manassas, VA). The transcription status of CHI3L1 in HEC1b cells was checked by RT-PCR as described above. The plasmid PLKO_AS3w.puro was purchased from the National RNAi Core Facility (Academia Sinica, Taiwan). To generate PLKO_AS3w CHI3L1.puro, CHI3L1 was first amplified by PCR using HEC1b cell cDNA as the template with sense primer (NheI) Hu-CHI3L1_S_NheI GGCTAGCACCATGGGTGTGAAGGCGTCTCAAAC and anti-sense primer (PstI) Hu-CHI3L1_AS_PstI TAACTGCAGCTCAGCCTGGGACTCAGCA. The amplified product was then cloned into the NheI/PstI sites of PLKO_AS3w.puro. The transfection was followed the protocol on the website http://rnai.genmed.sinica.edu.tw/webContent/web/protocols. HEC1b cells transfected with PLKO_AS3w.puro (mock) were used as the control. The CHI3L1 expression was assayed by RT-PCR and Western blot. The stable clones of various HEC1b transfectants were used in the following experiments.

### Knock-down of CHI3L1 in HEC151 cells

To eliminate endogenous CHI3L1 expression in HEC151 cells, an *in-vitro* knock-down experiment by small hairpin RNA (shRNA) targeting to CHI3L1 were performed. shRNA for CHI3L1 was purchased from National RNAi Core Facility. CHI3L1 shRNA constructed lentivirus were collected from 293T cells transfected with shRNA vector, Δ8.91and pMD.G. All plasmids were re-suspended in Opti-MEM® medium and transfected by using Lipofectamine2000 reagent according to the manufacturer's instructions. To generate CHI3L1-knockdown HEC151 cells (HEC151-shCHI3L1), HEC151 cell were seeded in 6-cm dishes and infected with CHI3L1 shRNA lentivirus containing 10 μg/ml polybrene for 48 hours. For selecting of stable clones, puromycin were added to the culture medium for further 48-hour incubation. The CHI3L1 expression was assayed by RT-PCR and Western blot. The stable clones of various HEC151 transfectants were used in the following experiments.

### Apoptotic assays of the ovarian cancer cells

Apoptotic assays of the various OVCAR3, CA5171, HEC1b and HEC151 transfectants treated with respective cytotoxic agent were performed by detecting 7-amino-actinomycin D (7-AAD) and annexin V in these cells. Briefly, the cells were treated with 2.5 nM of paclitaxel or 12.5 μM of cisplatin for 48 hours. Paclitaxel and cisplatin were purchased from Sigma-Aldrich (Sigma-Aldrich, St. Louis, MO). The preparation and dosage of these drugs were according to the manufacturer's instructions and previous studies [50, 51]. Cells treated with PBS were used as the negative control. The cells were then incubated with 7-AAD and FITC-conjugated annexin V (BD Bioscience, Franklin Lakes, NJ), respectively, according to the manufacturer's instructions, and were then analyzed by flow cytometry (FACScan; BD Bioscience, Franklin Lakes, NJ).

### Immunoprecipitation and immunoblotting

Immunoblotting assays of the various OVCAR3, CA5171, HEC1b and HEC151 transfectants were performed. Briefly, the cells were first lysed in the immunoprecipitation assay buffer, and the protein extracts were quantified using a BCA Protein Assay Kit (Pierce, Rockford, IL). Fifty μg of each cell lysate was then resolved by SDS/PAGE (12% gel), transferred onto a PVDF/nylon membrane (EMD Millipore, Billerica, MA), and probed with Abs specific to α-tubulin, YKL, Mcl-1, Bcl-2, Bak and Bax (*Upstate Biotechnology*, Lake Placid, NY). The membrane was then probed with horseradish peroxidase-conjugated secondary Ab (*Upstate Biotechnology*, Lake Placid, NY). The specific bands were visualized by an ECL® Western blotting system (GE Healthcare, Little Chalfont, UK). Protein levels were measured by densitometric analysis and normalized to the levels of α-tubulin (control) by ImageQuant 5.0 software (Molecular Dynamics Inc., Sunnyvale, CA). The expression level of each protein was presented as the fold change in comparison with the density of α-tubulin, and the expression levels in the original OVCAR3, CA5171, HEC1b and HEC151 cells were used as the reference.

### Statistical analysis

All statistical analyses were performed using SPSS version 15.0 (SPSS Inc., Chicago, IL, USA). Comparisons between unpaired groups were made using one-way analysis of variance (ANOVA) or the Student's *t*-test for continuous variables and Fisher's exact test for categorical variables. Levels of CHI3L1 of more than 0.1 were defined as a high CHI3L1 expression, and those less than 0.1 were defined as a low CHI3L1 expression. Survival curves were generated using the Kaplan-Meier method, and differences in survival curves were calculated using the log rank test. Cox regression analysis was used to evaluate the prognostic factors for recurrence and death. All data were expressed as mean ± S.E. from at least three different experiments. *p* values less than 0.05 were considered to be statistically significant.
